# Species‐habitat networks reveal conservation implications that other community analyses do not detect

**DOI:** 10.1002/eap.3073

**Published:** 2025-01-20

**Authors:** Zhaoke Dong, Andrew J. Bladon, Coline C. Jaworski, Richard F. Pywell, Ben A. Woodcock, William R. Meek, Peter Nuttall, Lynn V. Dicks

**Affiliations:** ^1^ College of Plant Health and Medicine Qingdao Agriculture University Qingdao China; ^2^ Department of Zoology University of Cambridge Cambridge UK; ^3^ Ecology and Evolutionary Biology Division School of Biological Sciences, University of Reading Reading UK; ^4^ Institut Sophia Agrobiotech (ISA) Université Côte d'Azur/INRAE Sophia‐Antipolis France; ^5^ UK Centre for Ecology and Hydrology Wallingford UK; ^6^ School of Biological Sciences University of East Anglia Norwich UK

**Keywords:** bumblebee, butterfly, calcareous grassland, interaction web, modularity, pollinators, restoration, specialization

## Abstract

Grassland restoration is an important conservation intervention supporting declining insect pollinators in threatened calcareous grassland landscapes. While the success of restoration is often quantified using simple measures of diversity or similarity to target communities, these measures do not capture all fundamental aspects of community reconstruction. Here, we develop species–habitat networks that aim to define habitat‐level foraging dependencies of pollinators across restored grassland landscapes and compare their value to these more conventional measures of community restoration. We assessed this across Salisbury Plain (UK), which represents the largest area of chalk grassland in northwestern Europe, encompassing six distinct management types aimed at the restoration and maintenance of species‐rich calcareous grassland. Sites that were previously disturbed or reverting from arable agriculture were comparable with those of ancient grasslands in terms of pollinator abundance and species richness. However, intensively managed grasslands exhibited notably lower values across nearly all measured indicators, including flower and pollinator richness and abundance, than ancient grasslands, with unmanaged grasslands following closely behind. This underscores the need for caution with both long‐term neglect and highly intensive management. Applying our species–habitat network approach, we found that pollinator communities in grasslands recovering from past military disturbance showed stronger modular associations with those in ancient grasslands than areas recovering from intensive agriculture. This highlights the importance of habitat history in shaping restoration trajectories. We propose that species–habitat networks should be part of the standard analytical toolkit assessing the effectiveness of restoration at landscape scale, particularly for mobile species such as insects.

## INTRODUCTION

Land use changes driven by agriculture have caused major biodiversity losses (Newbold et al., 2015). Specifically, the degradation or destruction of floristically rich grasslands has adversely affected insect pollinators (Powney et al., 2019). Ecological restoration is urgently needed to reverse biodiversity loss and restore ecosystem functions (Suding et al., 2015). A common approach in terrestrial ecosystems is to restore target vegetation to an “indigenous reference” community, thereby supporting the recovery of trophic levels (Bullock et al., 2011; Ockinger et al., 2018). However, many interventions' effectiveness in restoring ecosystems have seldom undergone quantitative assessment (Kaiser‐Bunbury et al., 2017). Bullock et al. (2022) recently proposed that enhancing ecological complexity itself—defined as the number of components in a system and connections among them—should be a restoration goal.

Pollinators are essential for maintaining terrestrial ecosystems and diverse agricultural food production (Ollerton, 2017). Nevertheless, they are declining rapidly due to various environmental drivers, including habitat loss, agricultural intensification, climate change, invasive species, and disease (Potts et al., 2010; Woodcock et al., 2016). Changes in land cover and configuration, land management, and pesticide use exert significant pressure in most regions (Dicks et al., 2021). The reestablishment of diverse pollinator assemblages has the potential to play an important role in wider ecosystem restoration.

Reestablishing mobile insect communities, such as pollinators, following the restoration of plant communities presents several challenges (Guiden et al., 2021). Firstly, the recolonization of many phytophagous invertebrates during restoration heavily relies on their dispersal ability, capacity to persist within nontarget habitats that dominate the wider landscape, and the presence of both suitable host plants and structural refuges in restored sites (Knop et al., 2011). Secondly, unlike plant communities, where the structure and composition of target communities are often well‐defined, restoration success for invertebrate communities is typically assessed by quantifying similarity to target communities or employing simpler diversity metrics (Woodcock et al., 2010; Woodcock et al., 2012). In a global meta‐analysis of terrestrial restoration studies, nearly half of the datasets measured biodiversity using taxonomic richness, with 61% focusing on plant communities (*N* = 608) rather than invertebrates (*N* = 280) (Atkinson et al., 2022). However, the response of plant and insect communities to the same management actions can vary dramatically (New et al., 2010; Swengel, 2001). Understanding the response of mobile insect communities to restoration is crucial from both conservation and ecosystem functioning perspectives, necessitating the development of appropriate methodologies (Knop et al., 2011).

When assessing restoration success for pollinator communities, patterns of plant–pollinator interactions can be compared between restored and reference sites (Campbell et al., 2019). Such a pollination network approach may better predict system complexity and response to change than simple measures of faunal diversity or similarity to target habitats (Bullock et al., 2022) and could therefore guide restoration management (Devoto et al., 2012). For instance, it has been shown that phylogenetically diverse vegetation can support complex trophic interactions with foraging pollinator communities, thereby enabling system persistence following restoration (Campbell et al., 2019).

Although plant–pollinator network approaches help understand community responses to environmental factors, they do not predict species resource use across multiple habitats. A species–habitat network approach develops bipartite networks where the nodes are species on the upper level and habitat patches or habitat types on the lower level, thus describing the spatial association between species and habitats in a particular landscape (Lami et al., 2021; Marini et al., 2019). This approach has been used to test the role of habitats in conserving invertebrate communities, including spiders (Nardi & Marini, 2021) and butterflies (Cappellari & Marini, 2021). It may also be used to measure restoration outcomes at a landscape scale by revealing species–habitat associations.

Calcareous grasslands have significantly declined due to agricultural intensification or abandonment threatening plant and insect pollinators of conservation concern associated with them (Habel et al., 2019; Ridding et al., 2015; van Swaay, 2002). Across Europe, strategies for restoring species‐rich seminatural grasslands are supported by various initiatives (Batáry et al., 2015). These strategies encompass grazing extensification, modified mowing practices, and the introduction of local provenance seeds through green hay spreading (Dicks et al., 2014).

Salisbury Plain Military Training Area covers ca. 38,000 ha of the Wiltshire Chalk and is one of the largest areas of continuous chalk grassland in northwestern Europe (Hirst et al., 2003). Fertilizer use, arable land conversion, and scrub encroachment resulting from grazing abandonment have all led to the degradation of grasslands on this site (Redhead et al., 2014). In this study, we quantified interactions between communities of plant‐ and flower‐visiting insects in a range of six grassland habitats typical of a gradient from improved, through restored to old‐growth calcareous grasslands on Salisbury Plain. We sought to understand how these communities reassemble in response to restoration management practices. We addressed the following hypotheses: (1) Recent intensive management or disturbance will reduce the species richness and abundance of insect pollinators when compared to ancient grasslands. (2) The complexity of plant–pollinator networks (e.g., connectance and generality) will vary across management types, with ancient grasslands exhibiting higher complexity than intensively managed and unmanaged grasslands. Restored grasslands will display intermediate levels of complexity depending on their restoration trajectory. (3) Species–habitat network analysis will reveal distinct habitat preferences for rare and specialist pollinator species, with stronger associations observed between ancient grasslands and specialist pollinators than other management types.

## MATERIALS AND METHODS

### Survey design

**FIGURE 1:**
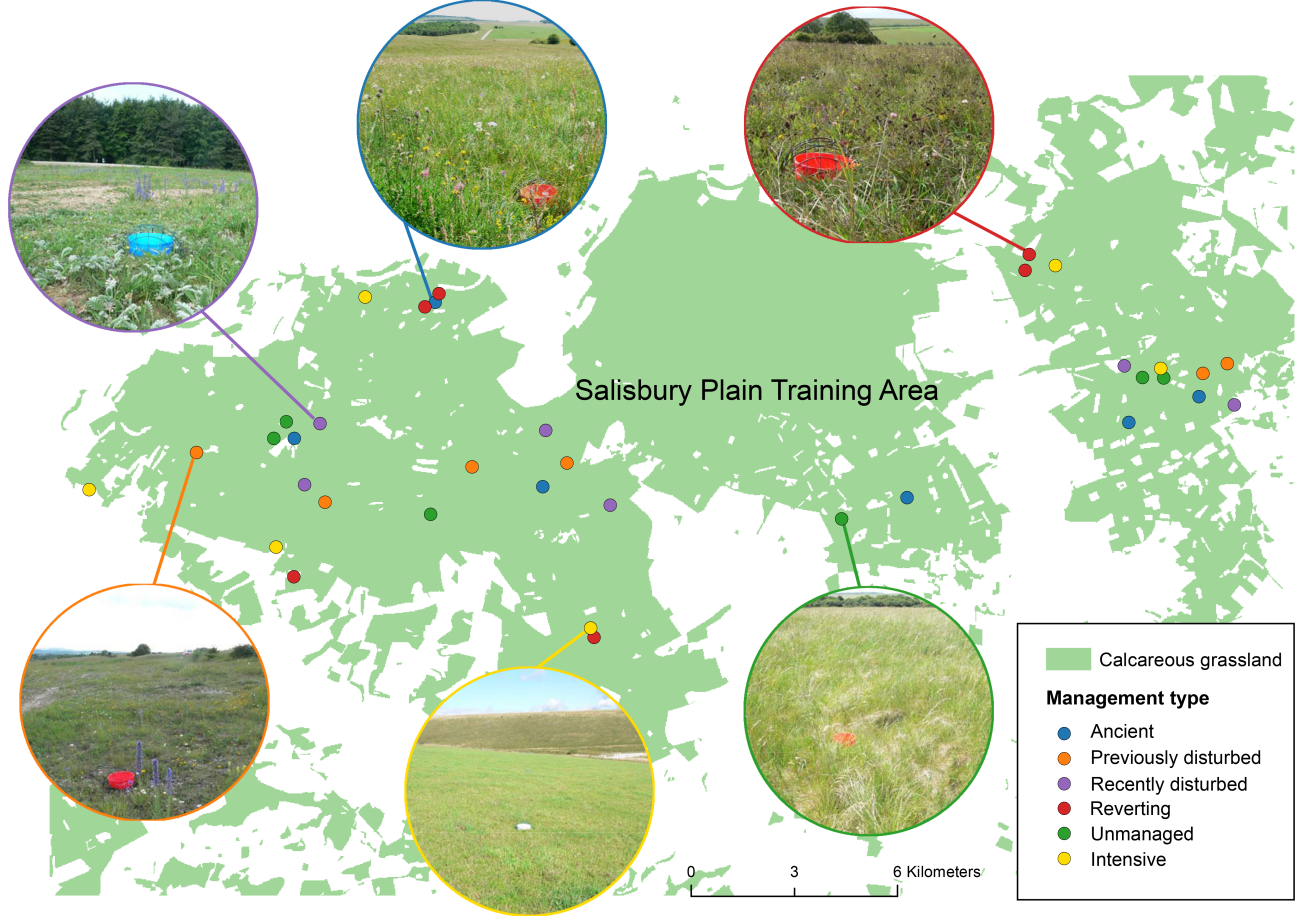
Location of the six distinct grassland habitats and their replicated monitoring sites across the Salisbury Plain Training Area. The map is based on data from the Land Cover Map of Great Britain 2017 (25 m rasterized land parcel dataset) produced by the Centre for Ecology and Hydrology. Photo credits: Richard Pywell.

The study was undertaken in the Salisbury Plain Training Area in Wiltshire, southern England (latitude 51°11′52″ N–51°16′4″ N; longitude 1°57′32″ W–2°9′32″ W). Plants, flower resources, and insect pollinators were recorded in six replicates of each of six distinct grassland habitats distributed across the Salisbury Plain Training Area (Figure 1). These habitats are expected to represent a gradient of plant species richness and flower resource provision comprising (1) lightly grazed, calcareous grassland that has remained extensively managed and been unimproved in living memory (Ancient); (2) lightly grazed, unimproved calcareous grassland previously disturbed by military training activities (>10 years ago) (Previously disturbed); (3) lightly grazed, unimproved calcareous grassland recently disturbed by military training activities (within last 3 years) (Recently disturbed); (4) lightly grazed, currently unimproved calcareous grassland reverting from arable use ca. 20–30 years ago (Reverting); (5) agriculturally improved, intensively grazed calcareous grassland (Intensive) (“improved” refers to manure added by penning animals, as opposed to inorganic fertilizers); (6) ungrazed, unimproved calcareous grassland (Unmanaged) (Table 1). Homogeneous c. 1‐ha patches of each distinct grassland habitat were selected for monitoring. These were situated within a matrix of mesotrophic and calcareous grassland communities.

**TABLE 1:** Descriptions of the six habitat types surveyed, in terms of the management practices used to restore or maintain them.

Management regime	No. of sites	Grazing	Fertilized	Military training disturbance
Ancient	6	Light	No	No
Previously disturbed	6	Light	No	>10 years ago
Recently disturbed	6	Light	No	<3 years
Reverting	6	Light	20 years ago	No
Intensive	6	Heavy	Yes	No
Unmanaged	6	No	No	No

### Sampling flower and flower–visitor interactions

In 2010, a permanently marked line transect (6 × 85 m) was established in the center of each habitat plot (36 in total). Due to uneven distribution of the managed grasslands, the transects were distributed alongside each type of grassland and did not strictly follow a random block design. Each of the 36 sites was sampled four times between 30 May and 19 September 2011, at approximately monthly intervals. Each transect was walked and lasted 15 min between 10:00 and 17:30 to coincide with the flight period of all major pollinating insects. This followed the method developed for the UK Butterfly Monitoring Scheme (BMS) (Pollard & Yates, 1993) and adapted as a standard method for bumblebee surveys (Pywell et al., 2005, 2006). Surveys were only undertaken when standardized environmental criteria were met (wind speed <5.5 m/s, not raining, temperature >17°C if cloudy or >13°C if less than 40% cloud cover). The shade (ambient) temperature, percentage sunshine, and wind speed were recorded at the end of each transect walk. It typically took 2 days to complete the transect counts for all 36 plots. During each survey, all butterflies (Lepidoptera: *Rhopalocera*) and day‐flying moths were identified to species level and counted. Foraging bumblebees (Hymenoptera: Apidae: *Bombus* sp.) were recorded to species level, following Prŷs‐Jones and Corbet (1991). Voucher specimens of rare species were collected for verification. Workers of *Bombus terrestris* and *Bombus lucorum* were collectively recorded as these cannot be reliably distinguished to species level in the field. In addition, summed counts were made for the following: honeybees, all solitary bees, and all hoverflies. The flowering plant that each bee or butterfly was first seen to visit was also recorded to species level. This enabled the construction of plant–pollinator interaction networks for each of the six replicated grassland types.

### Flower resources

During each visit to survey pollinators, flowers were counted in eight 0.5 × 0.5 m quadrats placed at equally spaced intervals along each transect. In each quadrat, all flowering dicotyledonous (broad‐leaved) plants were identified and the number of flower units for each species was counted. Flower units were defined as (a) single flowers (e.g., *Campanula rotundifolia*, *Helianthemum nummularium*, and *Gentianella amarella*), (b) multi‐flowered stems (racemes, corymbs) (e.g., *Anthyllis vulneraria*, *Onobrychis viciifolia*), (c) flower‐heads (capitulums) (e.g., *Leontodon hispidus*, *Centaurea scabiosa*, *Serratula tinctoria*), and (d) umbels (e.g., *Daucus carota*, *Pastinaca sativa*). These measures provided an estimate of the diversity and abundance of floral resources potentially available to pollinators through the season in each grassland habitat.

### Data analysis

We used four approaches to evaluate the effects of management types on plant and pollinator communities. All analyses were performed using R version 4.2.0 (R Core Team, 2022). We combined the data from four sampling sessions along the transect to provide a comprehensive overview of plant–pollinator interactions, which was utilized for species richness analysis of both plants and pollinators, as well as for analyzing the plant–pollinator network and implementing a species–habitat approach. The analysis of flower abundances and communities was based on quadrat counts of flower units, rather than pollinator interactions.

*Species richness and abundance.* We used generalized linear models (GLM) to assess the separate responses of plant and insect pollinator species richness to management type as a factorial predictor. Poisson errors and a log link function were used. The response of pollinator abundance to management type was also assessed using GLM with a negative binomial distribution to account for overdispersion (MASS package; Venables & Ripley, 2002). In cases where significant effects of management type were observed, we carried out post hoc pairwise comparisons between treatment levels by using Tukey comparisons (multcomp package; Hothorn et al., 2008). Rarefaction curves were generated using the Mao Tau function in the “vegan” package in R, with 100 randomizations to estimate species richness at increasing sampling efforts.

*Community composition*. Differences in plant species assemblages between the management types were tested with a permutational multivariate analysis of variance (PERMANOVA) (vegan package; Oksanen et al., 2022). We used the Bray–Curtis distance as the dissimilarity measure, as it is suitable for a variety of ecological data (Beals, 1984). Subsequently, we conducted pairwise post hoc tests using the package pairwiseAdonis (Martinez Arbizu, 2017). To visualize the variation in community composition across the six management types, we used nonmetric multidimensional scaling (NMDS) in the R package vegan. In order to evaluate the extent to which variations in flower and pollinator communities among different types of managed grasslands are influenced by either taxonomic turnover or nestedness (i.e., the extent to which communities with fewer species are a subset of richer ones), we computed the Sørensen dissimilarity index (beta.SOR; Baselga, 2010). beta.SOR was then decomposed into the Simpson dissimilarity index (beta.SIM), representing taxonomic turnover, and nestedness (beta.NES), indicating dissimilarity due to nested patterns within communities. All three indices were determined using the betapart package (Baselga, 2012). We conducted a Mantel test to assess the relationship between geographic distance and pollinator community dissimilarity across sites using 999 permutations. The test yielded a Mantel statistic 𝑟 = −0.0027 with a *p*‐value of 0.483, indicating no significant correlation between the spatial proximity of sites and the similarity of pollinator communities. This suggests that geographic distance does not have a discernible influence on the variation in pollinator community composition in our study area.

*Plant–pollinator network metrics*. Interaction data (pollinator visits to plants) were used to construct a plant–pollinator network for each of the 36 sites (Hypothesis 2). We excluded the hoverflies and solitary bees because they were not resolved to species level in the data. We used the “bipartite” package in R (Dormann et al., 2008) to calculate the five network metrics commonly used to represent community structure: (1) network size: the total possible links between pollinator species and flower species, (2) weighted connectance: the proportion of potential interactions that are realized, (3) pollinator generality: the number of flower species per visitor species, (4) plant generality: the number of visitor species per flower species, and (5) interaction evenness: the degree of homogeneity in interaction frequencies across the network. The network size was compared across management types using a GLM with a negative binomial distribution (following the same structure as described above) including flower abundance as a covariate. The other four metrics (connectance, pollinator generality, plant generality, interaction evenness) followed a Gaussian distribution and were tested in response to management types, network size, and flower abundance as covariates. Significance of the fixed effects was determined with an Anova function in the car package (Fox & Weisberg, 2019).

*Species–habitat network approach*. To test the associations between pollinator species and management types (Hypothesis 3), we pooled all the pollinator data from the 36 sites and grouped them based on the six management types. We built the network as a species–habitat pattern (Marini et al., 2019) to examine whether some species were more frequently found under particular management types across our whole dataset. We calculated two metrics that describe network structure: modularity and nestedness. Modularity measures the strength of division of a network into modules and indicates species preferences to specific habitats. Nestedness identifies nested modules in the network and provides information on species fluxes: If a network has a higher nestedness than expected by chance, this suggests that species‐rich habitats supply species to species‐poor habitats. The degree of both nestedness and modularity can have profound conservation implications (Martin et al., 2019). We chose weighted NODF (nested overlap and decreasing fill) (Almeida‐Neto et al., 2008) to calculate nestedness using the bipartite package (Dormann et al., 2008). Using the same package, we calculated modularity as well as the among‐module connectivity (*c*) and within‐module connectivity (*z*) of pollinators to identify species roles in the network. Before calculating these metrics, we removed the singletons and doubletons as well as any taxa not categorized at the species level (Dorado et al., 2011) (see Appendix S1: Table S1 for species list). Guimerà and Nunes Amaral (2005) suggested critical values of *c* lower than 0.62 and *z* higher than 2.5 for defining species' roles within the community, and Olesen et al. (2007) used these thresholds in pollination networks. Species exceeding either of these values were defined as generalists; that is, they linked to many habitats within their own module (high *z*, low *c*) or linked to several modules (low *z*, high *c*). Species with both a low *z* and a low *c* were peripheral species or specialists; that is, they had only a few links and always within their module (Olesen et al., 2007).

## RESULTS

### Flower resources

**FIGURE 2:**
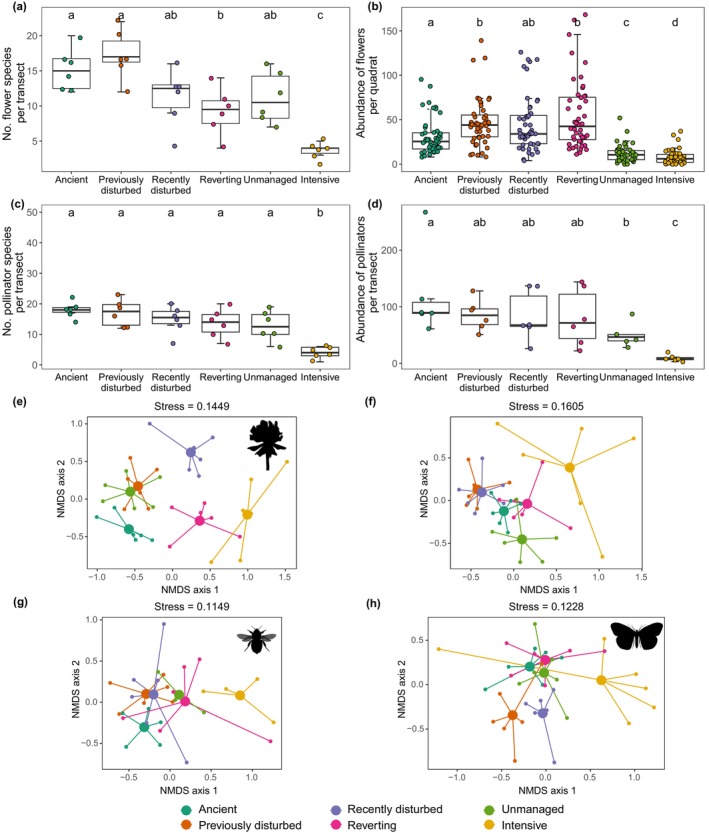
Diversity, abundance, and community structure of flowering plants and pollinators in the six types of grasslands differing by management: (a) species richness of flowers per quadrat, (b) abundance of flowers per quadrat, (c) species richness of pollinators per transect, and (d) abundance of pollinators per transect. Habitat types that do not share the same letter were significantly different from one another in post hoc pairwise comparisons. (e–h) Nonmetric multidimensional scaling (NMDS) ordination based on Bray–Curtis similarities of (e) flowers, (f) all pollinators, (g) bees, and (h) butterflies; each point represents an individual grassland; central circles are used solely for illustrative purposes. Image credits (all images downloaded from www.phylopic.org): *Bombus* by Melissa Broussard under an Attribution 3.0 Unported license; butterfly by T. Michael Keesey under a Public Domain Mark 1.0 license; flower by Andy Wilson under a CC0 1.0 Universal Public Domain Dedication license.

A total of 9479 flowers from 102 plant species were counted. The most abundant flowers were those of *Medicago lupulina*, *Lotus corniculatus*, *Hippocrepis comosa*, and *Onobrychis viciifolia*. There were significant differences in the flowering species richness, abundance, and community composition among the various management types (Figure 2a,b,e). The species richness of flowering plants was significantly higher in ancient grassland and previously disturbed grassland than in reverting and intensively managed grassland (Figure 2a). Intensively managed grassland had the lowest species richness among the studied grasslands. Flower abundance was significantly lower in the unmanaged and intensively managed grassland than in all other grasslands (LR Chisq = 49.130, df = 5, *p* < 0.001) (Figure 2b). The community composition of flowers exhibited remarkable distinctions across these grasslands (Figure 2e). The NMDS analysis yielded a stress value of 0.14. Additionally, the PERMANOVA indicated significant differences in community composition (*F*_5,30_ = 3.59, *p* = 0.001, *R* ^2^ = 0.37), as corroborated by pairwise adonis tests (all *p* < 0.05; Appendix S1: Table S2). The beta.SOR value of 0.69 indicates substantial dissimilarity in flower species composition among communities, driven primarily by species turnover (beta.SIM = 0.54), with a minor contribution from nestedness (beta.SNE = 0.15).

### Pollinators

A total of 2557 pollinators were recorded in the study sites, of which 51% were butterflies, 23% social bees, 16% moths, 10% hoverflies, and 1% solitary bees. This represented 46 species: 27 butterfly species, 7 moth species, and 12 bumblebee species (hoverflies and solitary bees were not identified to species). The shape of the rarefaction curves suggests that the sampling effort adequately represented the diversity of pollinators in the studied areas (Appendix S1: Figure S1).

Significant differences in pollinator species richness were observed across grassland management types. Intensively managed grassland had notably lower pollinator species richness (Figure 2c). Management significantly affected pollinator abundance (LR Chisq = 41.392, df = 5, *p* < 0.001), with ancient grasslands showing significantly higher pollinator abundance than unmanaged and intensively managed grasslands (Figure 2d). Intensively managed grasslands had the lowest pollinator abundance, while other grassland types showed similar abundances (Figure 2d). The NMDS analysis indicated a significant difference in pollinator community composition among the six management types (NMDS stress = 0.16, PERMANOVA: *F*_5,30_ = 3.56, *p* = 0.001, *R* ^2^ = 0.37). Notably, pollinator communities in ancient and reverting grasslands were statistically indistinguishable (pairwise adonis *p* > 0.05; Appendix S1: Table S3). Furthermore, pollinator communities in previously and recently disturbed grasslands did not differ strongly from each other, neither did those in previously disturbed and ancient grasslands (pairwise adonis *p* > 0.05; Appendix S1: Table S3). Bee communities differed significantly only between intensive and previously disturbed grasslands (pairwise adonis *p* = 0.045; Figure 2g). Butterfly communities also showed differences among the six management types (*F*_5,30_ = 3.57, *p* = 0.001, *R* ^2^ = 0.37), with similarity between ancient, reverting, and unmanaged grasslands (pairwise adonis *p* > 0.05; Figure 2h). Beta diversity analysis shows a relatively high beta diversity (beta.SOR = 0.51) among the different grassland management types, signifying substantial species composition dissimilarity. This dissimilarity is primarily driven by turnover (beta.SIM = 0.33), which contributes more significantly than nestedness (beta.SNE = 0.18) to the overall beta diversity.

### Plant–pollinator network metrics

We recorded a total of 2557 interactions in the sampling plots, excluding 251 interactions involving hoverflies and 19 involving solitary bees from further analysis. Network size varied significantly among the six management types (LR Chisq = 67.8, df = 5, *p* < 0.001), with intensively managed grassland having the smallest network size. Network size correlated with key metrics: negatively with weighted connectance (Pearson's correlation = −0.58, *p* < 0.001), positively with pollinator and plant generality (Pearson's correlation = 0.61 and 0.45, *p* < 0.001 and *p* = 0.005, respectively), but not with evenness (*p* = 0.9). When considering network size as a covariate, significant differences were found only in plant generality among management types, with previously disturbed grassland having higher plant generality than recently disturbed (post hoc *p* = 0.021; Table 2). While bipartite network structures exhibited similar complexity across ancient, previously disturbed, recently disturbed, and reverting grasslands, they diverged notably from those observed in unmanaged and intensively managed grasslands (Appendix S1: Figure S2a–f).

**TABLE 2:** Results from independent linear models with individual web metrics as response variables, and network size, flower abundance, and management type as predictor variables.

Response variable	Predictors	df	*F*‐value	*p*
Network size	Flower abundance	1	LR Chisq = 2.2	0.14
	Management type	5	LR Chisq = 86.7	**<0.001**
Connectance	Network size	1, 28	24.97	**<0.001**
	Flower abundance	1, 28	0.41	0.53
	Management type	5, 28	1.98	0.11
Interaction evenness	Network size	1, 28	0.26	0.61
	Flower abundance	1, 28	0.07	0.79
	Management type	5, 28	0.77	0.58
Pollinator generality	Network size	1, 28	13.12	**0.001**
	Flower abundance	1, 28	1.21	0.28
	Management type	5, 28	0.60	0.70
Plant generality	Network size	1, 28	5.75	**0.023**
	Flower abundance	1, 28	2.61	0.12
	Management type	5, 28	4.02	**0.007**

*Note*: Significant *p*‐values are shown in bold. Network size was assessed using a chi‐squared LR test due to its fitting by GLM with a negative binomial distribution.

Abbreviations: GLM, generalized linear models; LR, likelihood‐ratio test.

### Species–habitat network structure and habitat specialization

**FIGURE 3:**
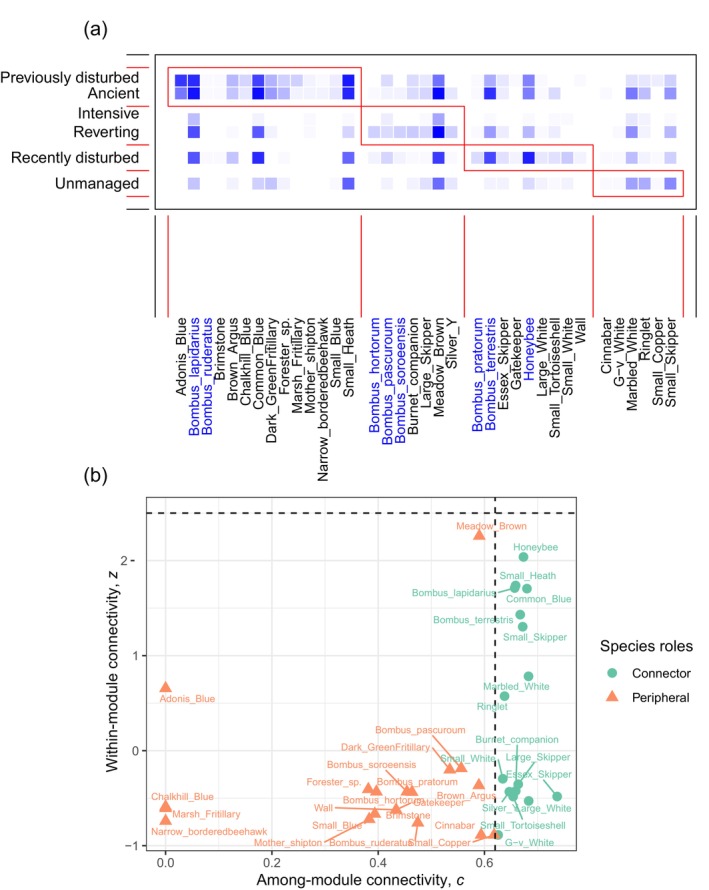
(a) Modules of the species–habitat network in 36 grasslands with six different management types. Habitat and pollinators are arranged in rows and columns, respectively. Red squares show the four modules identified by the analysis. Butterfly and moth species names are shown in black, and bees are shown in blue. (b) Pollinator generalism in the network based on among‐module connectivity, *c*, and within‐module connectivity, *z*, values. The dashed lines are the threshold values given by Olesen et al. (2007); see *Materials and methods* for details.

The species–habitat network (Appendix S1: Figure S2g) showed a moderate level of nestedness, as indicated by an observation NODF score of 0.33 (falls outside of the range of scores for randomization: −0.10 to 0.22). This nesting pattern suggests that the pollinator species used habitats in a nested way such that species found in species‐poor habitats are a subset of those in species‐rich habitats. The network exhibited a significant level of modularity (observed *z* = 23.65), exceeding two standard deviations from a random network. This suggests high habitat preference by pollinator species. We identified four modules (Figure 3a), each encompassing 6–14 pollinator species. Previously disturbed and ancient grasslands clustered within the same module, while intensively managed and reverting grasslands shared another module. Recently disturbed and unmanaged grasslands had their own distinct modules. It is noteworthy that bumblebee species were largely absent from the module associated with unmanaged grasslands.

Most pollinator species (75%) exhibited low within‐module connectivity (*z*) and among‐module connectivity (*c*) values (Figure 3b). This suggests they are specialists, primarily forming links within their respective modules. In contrast, 17 species were connectors, displaying low *z* values but high *c* values, signifying their role as habitat generalists. The module containing unmanaged grasslands was dominated by generalists, while the module containing ancient and previously disturbed grasslands consisted mostly of specialists. This included the four species with the lowest among‐module connectivity (Adonis Blue, Chalkhill Blue, Marsh Fritillary, and Narrow‐bordered Bee Hawk‐moth), the latter two of which are UK Biodiversity Action Plan Priority Species (BRIG, 2007). This suggests their specific adaptation to these plant‐rich habitats.

## DISCUSSION

This study demonstrates the complex recovery patterns of pollinator communities in restored calcareous grasslands, emphasizing the crucial role of habitat history. While basic diversity metrics and plant–pollinator network analyses revealed limited variation in the pollinator communities among management types, the species–habitat network approach unveiled key differences. Pollinator communities in grasslands recovering from past military disturbance exhibited stronger associations with those in ancient grasslands than areas recovering from intensive agriculture. This suggests that the type of past land use significantly influences the trajectory of pollinator community reassembly.

### Species richness and abundance across management types

Our first hypothesis proposed that the magnitude of restoration effects on pollinator communities would be influenced by the history of land use. This was supported by our findings, as pollinator communities varied among grasslands with different land use histories. For instance, while all restored grasslands exhibited similar levels of pollinator species richness and abundance to ancient grasslands, most of the restored grasslands hosted distinct pollinator communities. Only reverting grasslands shared similar communities with ancient grasslands. The history of management shapes the recovery trajectory of plant–pollinator communities in calcareous grasslands. These different trajectories may be due to the priority effect where initial colonizers slow later recoveries (Young et al., 2001, 2005). Human activities (e.g., grazing, fertilization, disturbance) can substantially alter plant communities. Restoring plant diversity and community composition comparable to ancient calcareous grasslands typically requires long‐term regeneration (Redhead et al., 2014). Although previously disturbed, recently disturbed, and unmanaged grasslands exhibited similar species richness to the target vegetation, their flower community composition remained distinct from ancient grasslands. This adds further evidence that calcareous grassland restoration is a very long‐term activity (requiring longer than 30 years), if the objective is to match the composition, rather than the species richness of the target plant community. Military training disturbance (previously/recently) did not cause species loss or flower abundance reduction in the present study, potentially due to low disturbance intensity. Strategic use of medium‐to‐low disturbance events can create short‐term and small‐scale heterogeneity in species composition and sward structure and achieve high‐quality diversity levels more quickly (Hirst et al., 2003, 2005).

Intensively managed grasslands exhibited notably lower values across nearly all measured indicators, including flower and pollinator richness and abundance, than ancient grasslands, with unmanaged grasslands following closely behind. This is valuable information for grassland conservation and management, indicating that both long‐term neglect and highly intensive management should be approached with caution. While management practices such as grazing and low‐to‐medium intensity disturbances can enhance biodiversity, overly intensive management appears to suppress pollinator diversity, as seen in the reduced species richness and abundance of intensively managed grasslands. Similarly, unmanaged grasslands, although less detrimental, may also fail to sustain pollinator communities at the same level as more optimally managed sites.

Taxa exhibited varying responses to management types, with bee communities differing only between intensively managed and previously disturbed grasslands. This may be attributed to our bee dataset mainly consisting of bumblebees (*Bombus* sp.), which are known to travel relatively large distances (>1 km) to find resources (Dicks et al., 2015). While they tend to prefer perennial and native plants with specific floral characteristics (Sikora et al., 2020), they are also generalist in their choice of floral resources and none are “oligolectic.” Most species of *Bombus* are also ground‐nesting and can nest in disturbed areas (Winfree et al., 2009).

### Plant–pollinator network structure across management types

The identity, diversity, and abundance of flowering plants within a habitat directly influence the composition and interactions within plant–pollinator networks, making network analysis a valuable tool for assessing restoration success (Motivans Svara et al., 2021; O'Connell et al., 2022). Our second hypothesis predicted that the complexity of plant–pollinator networks would vary across management types, with ancient grasslands exhibiting higher complexity than intensively managed and unmanaged grasslands, and restored grasslands displaying intermediate levels depending on their restoration trajectory. While we did observe significant differences in network size, with intensively managed grasslands having the smallest networks, other commonly used network metrics such as connectance, pollinator generality, and interaction evenness did not reveal clear patterns among the management types. This suggests that these metrics might not fully capture the nuances of network complexity and the effects of different management types on plant–pollinator interactions.

The lack of clear patterns in network metrics could be attributed to several factors. Firstly, these metrics often focus on quantitative aspects of the network, such as the number of interactions or the diversity of interactions, without considering the qualitative nature of those interactions. For example, two networks might have similar connectance values but differ in the specific species involved and the nature of their interactions, potentially obscuring important ecological distinctions. Secondly, different species can sometimes fill similar ecological roles, leading to similar network structures even when the species composition varies due to management types. The observed lack of clear patterns in network complexity metrics despite differences in species composition aligns with the findings from beta diversity analysis. Beta diversity analysis revealed that the dissimilarity in pollinator communities among the different management types is primarily driven by species turnover, indicating a substantial substitution or replacement of species. Nestedness, where species‐poor communities represent subsets of richer communities, played a lesser role in shaping these patterns. Therefore, evaluating restoration success requires looking beyond common network metrics and considering the specific habitat needs and ecological roles of individual pollinator species within the community.

### Insights from species–habitat network analysis

In our study, species–habitat networks were employed to compare pollinator communities in different management types of grassland. Species richness, abundance, and plant–pollinator network metrics did not uncover clear patterns among management types. For instance, ancient and reverting grasslands had similar pollinator communities, species richness, and abundance. However, when examining species–habitat network, both habitats were separated into two distinct network modules, revealing management effects on pollinator communities.

The species–habitat network module corresponding to recently disturbed grasslands encompassed mostly generalist butterfly species. Disturbance resulting from military training activities, such as vehicle traffic, can significantly impact plant composition (Hirst et al., 2003). This recently disturbed habitat hosted many annual dicotyledenous flowers that attracted widespread, mobile butterflies and bumblebees. In contrast, grasslands recovering from past disturbance had rich floral resources, supporting diverse pollinators, including rare butterfly species such as the Marsh fritillary (Botham et al., 2011).

## CONCLUSION

The findings of this study have important implications for grassland restoration efforts. Recognizing the influence of land use history can help set realistic expectations and tailor management strategies accordingly. The restoration of grasslands with a history of intensive agriculture may require more intensive interventions, such as planting native wildflowers or creating bare ground for ground‐nesting bees, to overcome the legacy effects of past land use and accelerate pollinator recovery. On the other hand, grasslands recovering from less intensive land uses, such as military disturbance, may require less intervention and exhibit faster recovery toward the target community composition.

This study sought to understand how pollinator communities reassemble in response to different management types in restored calcareous grasslands. Effectively evaluating and guiding grassland restoration requires moving beyond simple metrics and embracing a multifaceted approach that captures the complexities of community reassembly. Our study demonstrates the value of integrating traditional measures of diversity and network structure with the novel perspective offered by species–habitat networks. Species–habitat network analysis provides crucial insights into the habitat preferences and functional roles of pollinators, particularly mobile species, within restored landscapes. This perspective is important for pollinators, which are mobile and lack artificial restoration options (as is the case for plants that can be seeded). We recommend this technique becomes standard in restoration ecology, whenever restoration efforts are being planned at landscape scale and the target community includes mobile species operating at landscape scale, such as birds, mammals, or insects.

## AUTHOR CONTRIBUTIONS

Richard F. Pywell designed the study. Lynn V. Dicks and Zhaoke Dong designed the analytical methods. William R. Meek and Peter Nuttall undertook data collection. Ben A. Woodcock conducted data preprocessing and revised the manuscript. Zhaoke Dong performed the data analysis and wrote the first draft. Andrew J. Bladon and Coline C. Jaworski provided substantial revisions. All authors contributed substantially to the drafts and gave final approval for publication.

## CONFLICT OF INTEREST STATEMENT

The authors declare no conflicts of interest.

## OPEN RESEARCH STATEMENT

Data (Dong et al., 2024a) are available in Dryad at https://doi.org/10.5061/dryad.73n5tb33c. Code (Dong et al., 2024b) is available in Zenodo at https://doi.org/10.5281/zenodo.11136672.

## Supporting information


Appendix S1.
