# Supplementary material for: Species‐habitat networks reveal conservation implications that other community analyses do not detect
Source: Ecol Appl. 2025 Jan 20;35(1):e3073. doi: 10.1002/eap.3073 (PMC11744225; doi:10.1002/eap.3073)
Supplement: Supplementary file 1 — Appendix S1. [file EAP-35-e3073-s001.pdf]

Ecological Applications

Species-habitat networks reveal conservation implications that other community analyses do not detect

Zhaoke Dong, Andrew J. Bladon, Coline C. Jaworski, Richard F. Pywell, Ben A. Woodcock, William R. Meek, Peter Nuttall, Lynn V. Dicks

Appendix S1.

Table S1. List of pollinators, group information, abundance, and participation in species-habitat network modularity analysis. Note: Only species beyond singletons or doubletons, and those identified to the species level, were considered for modularity analysis. "Y" indicates participation; "N" indicates non-participation.

Code no.	Pollinator	Group	sum of abundance	whether participated in modularity analysis (Y/N)
1	*Bombus hortorum*	Bee	11	Y
2	*Bombus humilis*	Bee	1	N
3	*Bombus lapidarius*	Bee	242	Y
4	*Bombus muscorum*	Bee	2	N
5	*Bombus pascuroum*	Bee	26	Y
6	*Bombus pratorum*	Bee	9	Y
7	*Bombus ruderatus*	Bee	3	Y
8	*Bombus rupestris*	Bee	2	N
9	*Bombus soroeensis*	Bee	12	Y
10	*Bombus sp (Psithyrus)*	Bee	6	N
11	*Bombus terrestris*	Bee	135	Y
12	*Apis mellifera (Honeybee)*	Bee	126	Y
13	Adonis Blue	Butterfly	77	Y
14	Brimstone	Butterfly	3	Y
15	Brown Argus	Butterfly	36	Y
16	Burnet companion	Butterfly	24	Y
17	Chalkhill Blue	Butterfly	11	Y
18	Chimney Sweep	Butterfly	1	N
19	Cinnabar	Butterfly	3	Y
20	Common Blue	Butterfly	240	Y
21	Dark GreenFritillary	Butterfly	44	Y
22	Dingy Skipper	Butterfly	1	N
23	Essex Skipper	Butterfly	17	Y
24	Forester sp.	Butterfly	24	Y
25	G-v White	Butterfly	3	Y
26	Gatekeeper	Butterfly	3	Y
27	Large Skipper	Butterfly	25	Y
28	Large White	Butterfly	9	Y

Code no.	Pollinator	Group	sum of abundance	whether participated in modularity analysis (Y/N)
29	Latticed Heath	Butterfly	1	N
30	Marbled White	Butterfly	83	Y
31	Marsh Fritillary	Butterfly	10	Y
32	Meadow Brown	Butterfly	375	Y
33	Mother shipton	Butterfly	6	Y
34	Narrow borderedbeehawk	Butterfly	3	Y
35	Orange Tip	Butterfly	1	N
36	Painted Lady	Butterfly	1	N
37	Peacock	Butterfly	1	N
38	Red Admiral	Butterfly	1	N
39	Ringlet	Butterfly	36	Y
40	Silver Y	Butterfly	17	Y
41	Small Blue	Butterfly	8	Y
42	Small Copper	Butterfly	5	Y
43	Small Heath	Butterfly	221	Y
44	Small Skipper	Butterfly	71	Y
45	Small Tortoiseshell	Butterfly	16	Y
46	Small White	Butterfly	12	Y
47	Wall	Butterfly	3	Y
48	Burnet moths (*Zygaena* sp.)	Butterfly	320	N
49	Solitary bee	Solitary bee	19	N
50	Hoverfly	Hoverfly	251	N

Table S2. Multilevel pairwise comparison among flowering plant communities with management types. The adjustment methods include Holm (1979) ("holm"), Hochberg (1988) ("hochberg"), Hommel (1988) ("hommel"), Benjamini & Hochberg (1995) ("BH" or its alias "fdr"), and Benjamini & Yekutieli (2001) ("BY"). The Bonferroni correction was not used because it is dominated by Holm's method. The grey cell shows p value > 0.05.

	holm		BY		BH		hommel	
pairs	p.value	p.adj	p.value	p.adj	p.value	p.adj	p.value	p.adj
Previously disturbed vs Recently disturbed	0.010	0.048	0.008	0.031	0.008	0.009	0.012	0.023
Previously disturbed vs Intensive	0.002	0.028	0.002	0.017	0.003	0.005	0.004	0.016
Previously disturbed vs Reverting	0.004	0.048	0.004	0.020	0.005	0.006	0.003	0.012
Previously disturbed vs Unmanaged	0.001	0.015	0.001	0.017	0.004	0.005	0.001	0.007
Previously disturbed vs Ancient	0.020	0.048	0.019	0.068	0.021	0.023	0.010	0.020
Recently disturbed vs Intensive	0.004	0.048	0.001	0.017	0.003	0.005	0.003	0.012
Recently disturbed vs Reverting	0.026	0.048	0.033	0.110	0.041	0.041	0.023	0.023
Recently disturbed vs Unmanaged	0.004	0.048	0.002	0.017	0.001	0.005	0.003	0.012
Recently disturbed vs Ancient	0.005	0.048	0.003	0.019	0.001	0.005	0.003	0.012
Intensive vs Reverting	0.006	0.048	0.005	0.023	0.004	0.005	0.001	0.007
Intensive vs Unmanaged	0.005	0.048	0.008	0.031	0.003	0.005	0.002	0.010
Intensive vs Ancient	0.005	0.048	0.003	0.019	0.003	0.005	0.003	0.012
Reverting vs Unmanaged	0.005	0.048	0.004	0.020	0.003	0.005	0.002	0.010
Reverting vs Ancient	0.006	0.048	0.002	0.017	0.002	0.005	0.002	0.010
Unmanaged vs Ancient	0.002	0.028	0.002	0.017	0.002	0.005	0.002	0.010

Table S3. Multilevel pairwise comparison among pollinator communities with management types. The adjustment methods include Holm (1979) ("holm"), Hochberg (1988) ("hochberg"), Hommel (1988) ("hommel"), Benjamini & Hochberg (1995) ("BH" or its alias "fdr"), and Benjamini & Yekutieli (2001) ("BY"). The Bonferroni correction was not used because it is dominated by Holm’s method. The grey cell shows p value > 0.05.

	holm		BY		BH		hommel	
pairs	p.value	p.adj	p.value	p.adj	p.value	p.adj	p.value	p.adj
Previously disturbed vs Recently disturbed	0.013	0.056	0.015	0.057	0.013	0.015	0.018	0.036
Previously disturbed vs Intensive	0.005	0.050	0.001	0.025	0.002	0.009	0.006	0.024
Previously disturbed vs Reverting	0.007	0.056	0.006	0.035	0.003	0.009	0.004	0.022
Previously disturbed vs Unmanaged	0.003	0.039	0.007	0.035	0.009	0.012	0.002	0.016
Previously disturbed vs Ancient	0.017	0.056	0.021	0.075	0.015	0.016	0.017	0.034
Recently disturbed vs Intensive	0.002	0.030	0.007	0.035	0.008	0.012	0.007	0.024
Recently disturbed vs Reverting	0.006	0.054	0.006	0.035	0.003	0.009	0.008	0.024
Recently disturbed vs Unmanaged	0.003	0.039	0.003	0.030	0.003	0.009	0.003	0.021
Recently disturbed vs Ancient	0.008	0.056	0.003	0.030	0.006	0.011	0.006	0.024
Intensive vs Reverting	0.012	0.056	0.009	0.037	0.010	0.013	0.012	0.027
Intensive vs Unmanaged	0.003	0.039	0.003	0.030	0.004	0.010	0.004	0.022
Intensive vs Ancient	0.002	0.030	0.001	0.025	0.002	0.009	0.003	0.021
Reverting vs Unmanaged	0.009	0.056	0.008	0.036	0.008	0.012	0.008	0.024
Reverting vs Ancient	0.125	0.125	0.131	0.435	0.122	0.122	0.121	0.121
Unmanaged vs Ancient	0.009	0.056	0.006	0.035	0.005	0.011	0.009	0.027

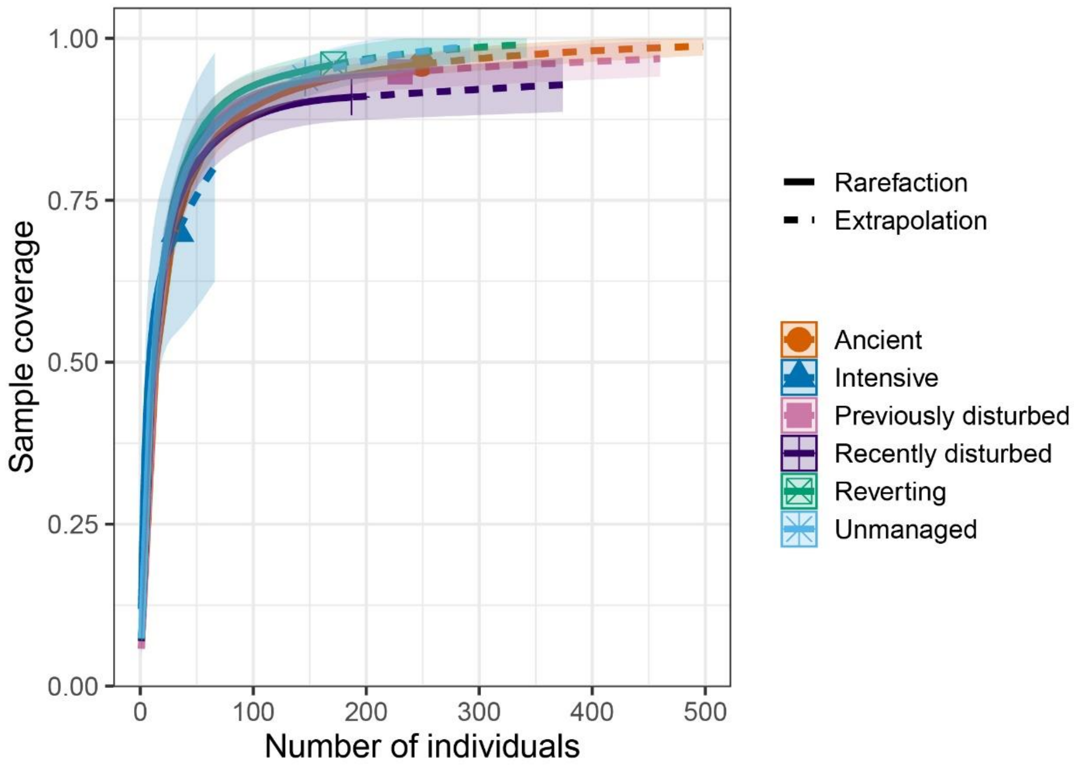

Figure S1 Rarefaction curves based on 1000 randomizations for the pollinators in the six management types

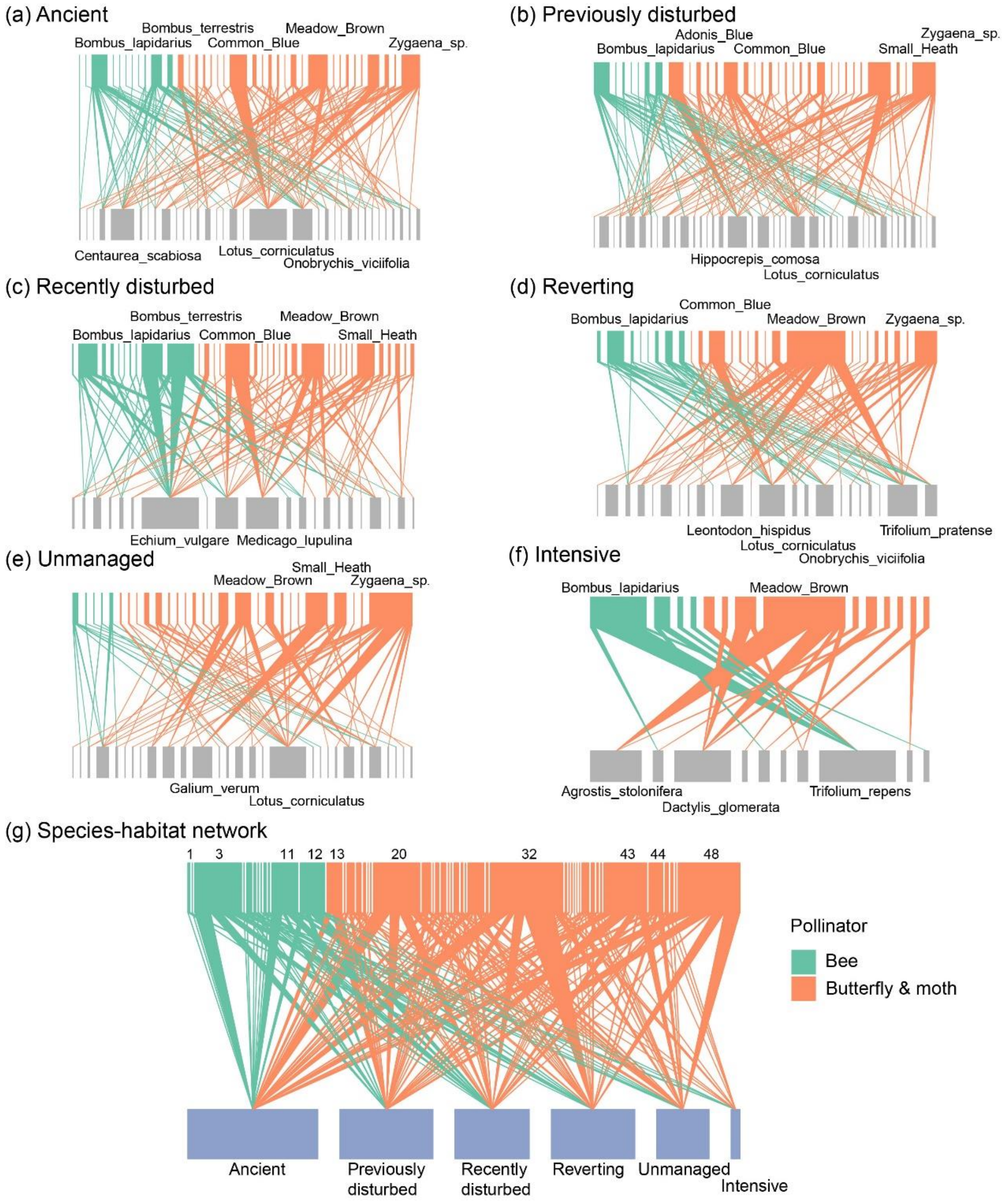

Figure S2 Bipartite quantitative network of interactions (links) between pollinators (top) and flower species/habitats (bottom). (a ~ f) flower-pollinator networks for the six management type grasslands. Different pollinator groups are represented by different colours. Each bottom rectangle represents a flower species. Width and the size of the links show the interaction frequency. Only some dominant species are shown. (g) species-habitat network. Each bottom rectangle represents a management type of grasslands. The coded number on each top rectangle refers to pollinator species, which details can be seen in table S1.

References

Benjamini, Y., and Y. Hochberg. 1995. Controlling the False Discovery Rate: A Practical and Powerful Approach to Multiple Testing. Journal of the Royal Statistical Society: Series B (Methodological) 57:289-300.

Benjamini, Y., and D. Yekutieli. 2001. The Control of the False Discovery Rate in Multiple Testing under Dependency. The Annals of Statistics 29(4):1165–1188.

Hochberg, Y. 1988. A sharper Bonferroni procedure for multiple tests of significance. Biometrika 75(4)800–802.

Holm, S. 1979. A Simple Sequentially Rejective Multiple Test Procedure. Scandinavian Journal of Statistics 6(2):65–70.

Hommel, G. 1988. A stagewise rejective multiple test procedure based on a modified Bonferroni test. Biometrika 75(2):383–386
